# The propensity to have children in Hungary, with some examples from other European countries

**DOI:** 10.3389/fsoc.2022.1009115

**Published:** 2022-12-02

**Authors:** Éva Berde, Áron Drabancz

**Affiliations:** Department of Economics, Corvinus University of Budapest, Budapest, Hungary

**Keywords:** decreasing total fertility rate, reproduction rate, economic policy measures, Becker-type quantity quality change, aging societies

## Abstract

In most countries around the world, the total fertility rate (TFR) has been on a downward trend over recent decades. In the developed, and many less developed countries this has led to the TFR being consistently below the replacement level of 2.1 over the long term, leading to population decline in the absence of immigration. Many European governments, including that of Hungary, are spending a significant proportion of GDP on family support to prevent fertility decline. Despite these efforts, we have not seen any significant increase in the TFR. In this article, we explain the decisions of families not to have children by using a more stringent formulation of Becker's original quantity-quality trade-off. We point out that increasing family support expenditure has not achieved its goal. While huge financial effort has been made to increase fertility rates, insufficient attention has been paid to alleviating the burden of the growing elderly population.

## Introduction

In the nineteenth and twentieth centuries, the rapid growth of Earth's population led many authors to make pessimistic predictions. For example, Malthus (1798) believed that while the population would increase quadratically over time, available resources would only increase linearly. According to Malthus, this would lead to ever greater impoverishment because the population would continue to grow. Looking at more recent writers, Ehrlich (1968) expressed similar thoughts, saying that humanity had lost the battle to sustain itself and that we could soon expect the destruction of civilization.

Authors who earlier predicted the demise of humanity assumed that Earth's population would continue to grow at an accelerating rate. Indeed, Earth's population grew from 1 billion to 2 billion in 123 years, while it took 33 years to reach 3 billion, and then only 14, 13, and 12 years to increase to the next billions. In 1999, the Earth's population was 6 billion (Bloom et al., 2003). However, this was also a turning point, because facts and estimates suggest that it will take an increasing amount of time for the population to grow by increments of another 1 billion (12, then 14, and 21 years). The population is expected to reach 10 billion in 2058 (United Nations, 2022). Further projections are increasingly uncertain, but the United Nations (2022) does not expect the population to reach even 10.5 billion by the end of the century. Vollset et al. (2020) expect the global population to decline from the last third of the century, and the United Nations (2022) also predicts a slight population decline by 2100. Overall, there have been substantial changes in the lifestyles of people of childbearing age in both developed and developing countries which, among other factors, clearly involve a preference for having fewer children. This will result, after a few decades, in population decline.

The fact is that, in about two-thirds of the world today, the problem is no longer population growth but population decline, either now or in the near future. Population decline and increasing life expectancy are associated with population aging, which is especially the case in more developed countries (Bloom et al., 2010). In many parts of the European continent, and elsewhere such as in East Asia, the threat of depopulation already looms. In the countries of Central, Eastern and Southern Europe, the impact of population decline is already being felt because, on the one hand, the Total Fertility Rate (TFR) has been below replacement level^1^ for a relatively long time, and on the other, there is a high level of emigration from many countries in the region of mainly the younger members of the population (Atoyan et al., 2016). This process further reduces the number of children that are born.

In those European countries (like France and United Kingdom; see the Worldometer database) where no population decline has yet occurred, the TFR has also fallen to low levels. In these countries, lower mortality rates and immigration have partially prevented population decline (Parr, 2021), and insufficient time has elapsed since the TFR fell below the replacement rate for the effect to be noticeable (Goldstein and Cassidy, 2021). In most African countries, in which the TFR remains well above the replacement rate, the relatively high TFR represents a decline from historical levels. In developing countries, the TFR averaged six in 1950 but had fallen to three by the early 2000s (Malmberg et al., 2006). In the early 2020s, the global TFR of 2.31 is barely above the level needed for reproduction. In addition, at least two-thirds of the world's population already live in countries where the total fertility rate is lower than the fertility rate needed for population reproduction (authors' calculation based on United Nations, 2022 data). According to United Nations' (2022) data, global population growth is still expected between 2020 and 2050, but about two-thirds of this growth will take place in Africa.

The fundamental cause of population decline is the fall in the TFR to below 2.1, the current replacement rate, which has only been offset by immigration in some countries. Goldstein and Cassidy (2021) have shown that at current mortality rates, and without immigration, it will take about 40 years from the time the TFR falls below replacement level for the population to start declining, assuming that the TFR remains at that low level. For this reason, many governments are using economic policies to increase the propensity of families (couples) to have children: They support parents by providing financial benefits and childcare services. It is however unclear how effective these measures are, and it is very difficult to investigate this question due to potential delays in the births of additional children following these measures, and the effects of random events. In addition, some measures may bring forward the birth of children that their parents had intended to schedule for later but may not result in the birth of additional children overall. In such cases, these measures will only temporarily increase the TFR, and their impact will only be realistically seen after 5–10 years. In this article, we do not attempt to provide an econometric analysis of the relationship between rapidly changing economic policy regulation and the TFR, but simply describe how expenditure on child support has evolved in the countries of relevance to us, especially Hungary, and how the TFR has changed over time. We draw our conclusions by describing the facts.

In the “Explanations of low fertility rates in the literature” section, following the introduction, we show how the literature since Becker (1960) has explained the declining preference for childbearing. Then, in the “Some examples of measures taken to increase fertility rates – a Hun-garian focus, and a European approach” section, we describe the changes in family-support policies and the evolution of the TFR in Hungary. The Hungarian trends are compared both with the situations in the Central and Eastern European countries that joined the European Union and with the situations in other European countries. In the “Explaining the even greater decline in fertility rates than before – in the spirit of Becker section, we modify the original model of Becker (1992) and theoretically explain the drastic decline in the TFR. Finally, in the “Conclusion” section, we describe our conclusions.

## Explanations of low fertility rates in the literature

Becker (1960) showed that the decision to have children can be viewed in the same way as the decision to obtain other ordinary goods. In terms of the number of children, quantity and quality may be seen as substitutable. Women are giving birth to fewer and fewer children but trying to provide their children with good living conditions and good educational opportunities. This idea is now commonplace in economics, and the decision to have children is often examined on this basis. Thus, deciding between quantity (i.e., number of children) and quality (what conditions and education to provide for children) has become central to the “consumption” of children as a good (see some of the most commonly cited articles that deal with this topic: Ben-Porath, 1973; Schultz, 1973; Becker and Lewis, 1974; Rosenzweig and Evenson, 1977; Blake, 1981; Becker, 1986, 1992; Doepke, 2005; Angrist et al., 2010).

In addition to economic considerations, we often encounter both ethical and philosophical approaches to childbearing. Häyry (2004) describes how rational arguments can be found for many conflicting reproductive theories. One of the most notable of these is the Old Testament revelation that conception is a gift from God. On this basis, many theologians hold that human beings must always accept God's gift and should not control the conception of a child (Clark, 1969). This idea is not confined to Christianity, and may be found in the views of members of other religions. Zhang (2008) argues that the issue of religion in childbearing is important because couples now have the option to prevent pregnancy, or in some cases choose abortion. However, most religions encourage their followers to marry rather than cohabit and do not interfere with the possibility of conception. Zhang (2008) also points out that religions are becoming more permissive, and it is perhaps for this reason that the fertility rates of religious and non-religious families are converging in most countries, leading to declines in fertility rates. This can also be explained by the fact that religiousness has undergone a major transformation, where according to theories about secularization, modernization processes have made negative impacts on the stability and strength of religious communities, and their practices and beliefs (Pollack, 2008). For many people, being religious is now different from what it meant for their parents: more and more people are abstaining from formal religious practice, rarely going to church or absenting altogether. They no longer consider themselves bound by religious observance (Davie, 1994; Aarts et al., 2008). The number of people who continue to practice their faith is gradually decreasing, especially in Europe (Molteni and Biolcati, 2018). As a result, the dogmatic approach of the church to childbearing and childrearing is having less influence (Molteni and Biolcati, 2018). Without a doubt, the prevailing religious norms, except religious extremism, have been fundamentally shaped by socioeconomic circumstances, and as these circumstances have changed so have the standards.

Caldwell (1978) showed that in developing economies that are adopting capitalism young people who choose wage employment are increasingly likely to have sufficient income to maintain a separate household and do not need to live in a traditional extended family to secure their livelihoods. The direction of cash flow between parents and children has also often reversed. New ways of life no longer require having many children. Similar ideas were expressed by Notestein (1953), who pointed out that the factors which have reduced the mortality rate have also reduced the need to have numerous children.

Bongaarts (1982) shows that fertility can be explained almost entirely by four variables: the proportion of married women in the population, contraceptive use among women, the number of abortions, and postpartum infertility. The first three of these four factors are a function of personal choice, involving a clear Beckerian quantity-quality trade-off and an increasing role for quality.

In addition, the role of children in the family has changed considerably over the last centuries. In the eighteenth and nineteenth centuries, children were often seen as a means of production from a young age, taking on a share of the family's household tasks. The lower level of mobility, the close intergenerational household structure, and, most importantly, the lack of a pension system, meant that children were also a guarantee of a secure old age for their parents. Children ensured the survival of their parents in adulthood, after a significant decline in the latter's working capacity.

Changes in living conditions have made this old lifestyle obsolete for the new generations, and the current situation for women is not conducive to having many children. McDonald (2006) also points out that the improved equality of women and incoherence in the approaches of social institutions are barriers to having more children. Although the institutions of modern society generally treat women as individuals in accordance with the principle of gender equality, the institutions that deal with the family support only a low level of gender equality.

Lutz (2007) explains that low fertility rates create a trap and prevent the number of children from increasing. When the TFR has been low for several decades, the number of women of childbearing age will eventually start to decline, thus even if the TFR increases to a high level the total number of children that are born will be less. In addition, the economic cause for the decline in fertility rates is based in the high aspirations of the younger generation, given the relative prosperity of their parents, but their earning potential is steadily declining, partly because of an aging society. In addition, the ideal number of children that the younger cohort desire is shrinking, as they themselves have grown up in smaller families.

In addition to this, Lutz (2005) show that the so-called “tempo effect” also greatly reduces fertility rates. Women are having their first children later, and sometimes waiting longer between children than before. This leads to a decline in women's ability to conceive.

**Figure 1:**
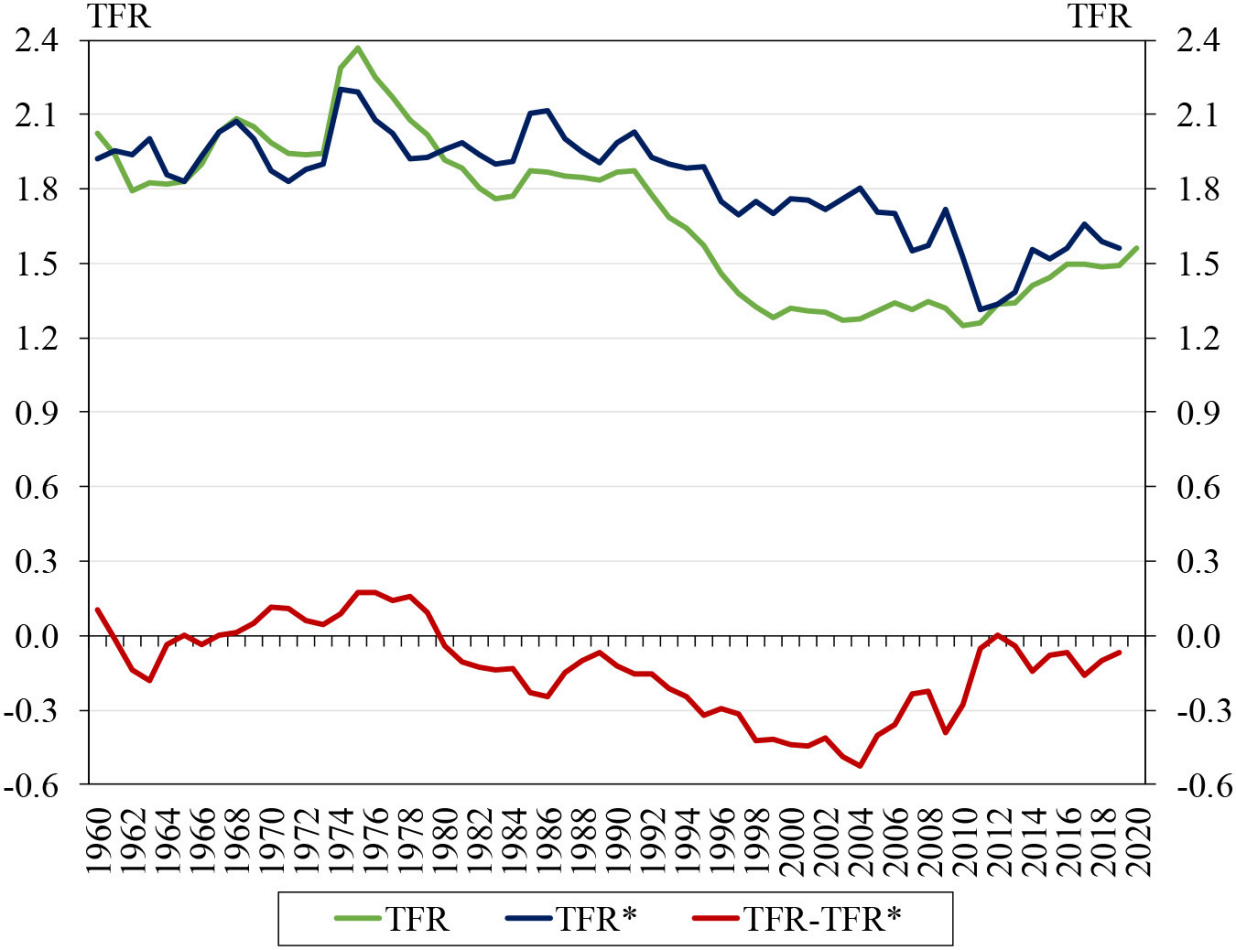
Evolution of the total fertility rate, the tempo-adjusted fertility rate, and the difference between the two, 1960–2020. Source: HFD (2022), authors' editing. TFR, total fertility rate; TFR^*^, tempo-adjusted total fertility rate.

Historically, Hungary has been ahead of other European countries in terms of its decline in the TFR. As Spéder and Kamarás (2008) noted, Hungary was the first country in Europe to witness a period wherein the total fertility rate was below the replacement level of 2.1 (in 1960), after which the TFR temporarily increased on multiple occasions, but as a result of further decreases has remained below the replacement level for all but 4 years since. Kamarás (2010) provides a comprehensive picture of childbearing trends in the 1990s and early 2000s. He clearly indicates that the increase in maternal age (see later under Figure 1 the description of the age development of mothers at the time of first birth), modifications in marriage vs. cohabitation patterns, and changes in couples' preferences have led to the aging of society and a decline in the Hungarian population. Kamarás (2010) also suggests that if the above factors do not change radically, further aging and even faster population decline can be expected in Hungary.

After serious economic policy efforts aimed at increases, the Hungarian TFR in 2020 was 1.56, according to the Hungarian Central Statistical Office, which is slightly above the average of 1.5 for EU countries. This comparatively ‘not so low’ TFR is also worth evaluating because, according to Spéder and Kapitány (2014), the probability of women realizing their intention to have children is significantly lower in post-communist countries than in Western European countries. However, this value still means that Hungary is not free of the problems caused by a low fertility rate.

Generally, on the European continent, children are no longer indispensable as a form of support for elderly parents. In addition, with improvements in nutrition, public health, and medical science, the probability of child survival has increased greatly in both developed and less developed countries. Thus, it is no longer necessary to give birth to such a large number of children to ensure that a sufficient number live to adulthood. The twentieth and twenty-first centuries have overall seen both a reduction in the necessity for older parents to be supported by their children and a reduction in the necessity for prolific childbearing to ensure a sufficient number of adult children. In addition, it has become possible to regulate conception, and parents may now opt to pursue a “quality” approach to children rather than one of quantity.

## Some examples of measures taken to increase fertility rates – a Hungarian focus, and a European approach

The fertility rate has been declining in Hungary since 1960, except for a few periods of brief increase; more importantly, as we have noted in the previous section, there have been only four occasions between 1960 and 2020, see Figure 1 below, when the fertility rate exceeded 2.1. During this period, increasing the fertility rate has become a national economic objective several times, both before and after the regime change. To this end, a number of major measures and many smaller ones were taken.

Paid maternity leave extending to 1 month after childbirth was introduced for mothers in industrial jobs as early as the late nineteenth century, but the duration thereof was increased during the era of socialism, and the benefit was made equal to the mother's previous salary. Every working woman became entitled to this benefit (Szikra and Szelewa, 2010). However, other forms of family support were introduced only later. In 1967, the enactment of the ‘Child Care Allowance’ (popularly known by Hungarian abbreviation ‘GYES’) was introduced (Oláh, 2003), which allowed mothers to stay with their children until the age of three without losing their employment, and with modest remuneration. In 1985, another form of maternity leave, the ‘Child Care Fee’ (‘GYED’), was enacted, which gave mothers about three-quarters of their previous salary for 1 year in exchange for caring their children (Oláh, 2003). This was later extended to when the child reached the age of two, and even later, some employment was allowed in addition to receiving GYED. Although the introduction of GYES and GYED did raise the TFR for a few years after a slight delay, the increase was never very large and after a few years, there was another decline. In consequence, Hungary's population fell from 10,200,298 in 2001 to 9,769,526 in 2020, according to the Hungarian Statistical Office.

The second type of measure is the so-called ‘baby loan’, known before the regime change as the ‘social housing subsidy’. The baby loan is a way of providing parents who have or will have children with support for the purchase of a house, a contribution to renovations, and a loan on very favorable terms. These grants were initially basically only available to families with at least three children, based on a Council of Ministers' decision in 1970, and were then significantly extended in 1985 by a Decree of the Council of Ministers. After many changes, sometimes involving the tightening up of this opportunity, the most generous version was introduced 2 years ago – the Prenatal Baby Support Loan. This loan makes it extremely easy for families to access housing.

The third type of measure is the so-called 'family allowance', which also has a very long history. This money provided to parents to support their children is not dependent on the parents' financial situation. The fourth measure is the tax decrease on earned income that parents are eligible for, depending on the number of children they have. This form of support was introduced a few years ago.

The budget for these types of benefits for parents with children gradually increased following the change of regime, with the exception of 1995. At that time, the economic austerity package, which was signed by the then Finance Minister Lajos Bokros, also reduced the support for parents with children, although that reduction proved to be temporary.

Despite all the support distributed in the years following the change of regime, the fertility rate fell even more sharply to 1.28 in 1999, and that decline continued later. After a slight rise, the TFR had fallen below 1.3 in 2010, and in 2011 it reached its lowest value ever, at 1.23. The economic crisis of 2008–2009 certainly contributed to these two extremely low values, but these levels still fit the underlying trend. Thus, in the early 2010s achieving a population turnaround once again became a national strategic objective. In 2018, the target was set of reaching a total fertility rate of 2.1 by 2030^2^. This has led to an increase in family-support expenditure as a share of GDP. According to the OECD (2022), 3.5% of GDP was already being spent on family support in Hungary in 2017, with only France having a higher share among OECD countries. In addition to the increase in the amount of family support, the family-support framework has also been restructured, with the share of direct, subject-to-children transfers (e.g., family allowance) decreasing, while the share of indirect, mostly favorable transfers linked to loans has increased (generally in the form of the so-called baby loan). Transfers associated with employment and wages (e.g., tax allowances) have also been increased. It should be noted that some subsidies, such as housing loans, have also had undesirable consequences: it is likely that childbearing subsidies are part of the reason why house prices have risen significantly. The overall availability of housing for smaller families and those not receiving subsidies has been on a downward trend since 2015 (Magyar Nemzeti Bank, 2022).

Figure 1 shows that from 2012 onwards the TFR did indeed start to increase. This might lead us to conclude that the increase in the total fertility rate in Hungary over the last decade is the result of changes in the family support system and an increase in expenditure, which raised the TFR to 1.56 in 2020.

However, by inspecting the graph for the tempo-adjusted total fertility rate (abbreviated TFR^*^), we can reach a different conclusion. The tempo-adjusted total fertility rate takes into account children who would have been born if women's childbearing age had been the same as before (Bongaarts and Feeney, 1998). We can see that in the period 2011–2020 the TFR^*^ barely exceeded the TFR, whereas previously the difference between the two indicators was quite large, even surpassing 0.4 in some years. This can be seen in the graph of the differences in Figure 1. The values of the TFR^*^ that exceed the TFR indicate women's postponement behavior (Sobotka, 2004), whereby they give birth to planned children at a later age, which is particularly true for the first child. When the period of recuperation (the time when they begin to give birth to the postponed children) begins, the TFR gradually reaches the level of the TFR^*^. We believe that this is what happened in Hungary in the 2010s. This claim is supported by the fact that, according to HFD (2022), the average age of mothers at the time of the birth of their first child was 25.1 years in 2000, 27.67 in 2010, and 28.36 in 2020.

This should raise doubts about the effectiveness of economic policy measures, since the fertility rate in Hungary increased by only 0.33 between 2011 and 2020. As Spéder (2021) points out, only the share of families with fewer children increased, and the cohort-specific live birth rates of younger cohorts did not. An increase of 0.33 was far from sufficient to bring fertility rates up to the replacement level of 2.1. As we have already written, Goldstein and Cassidy (2021) show that the persistence of low fertility rates over four decades results in population decline.

**Figure 2:**
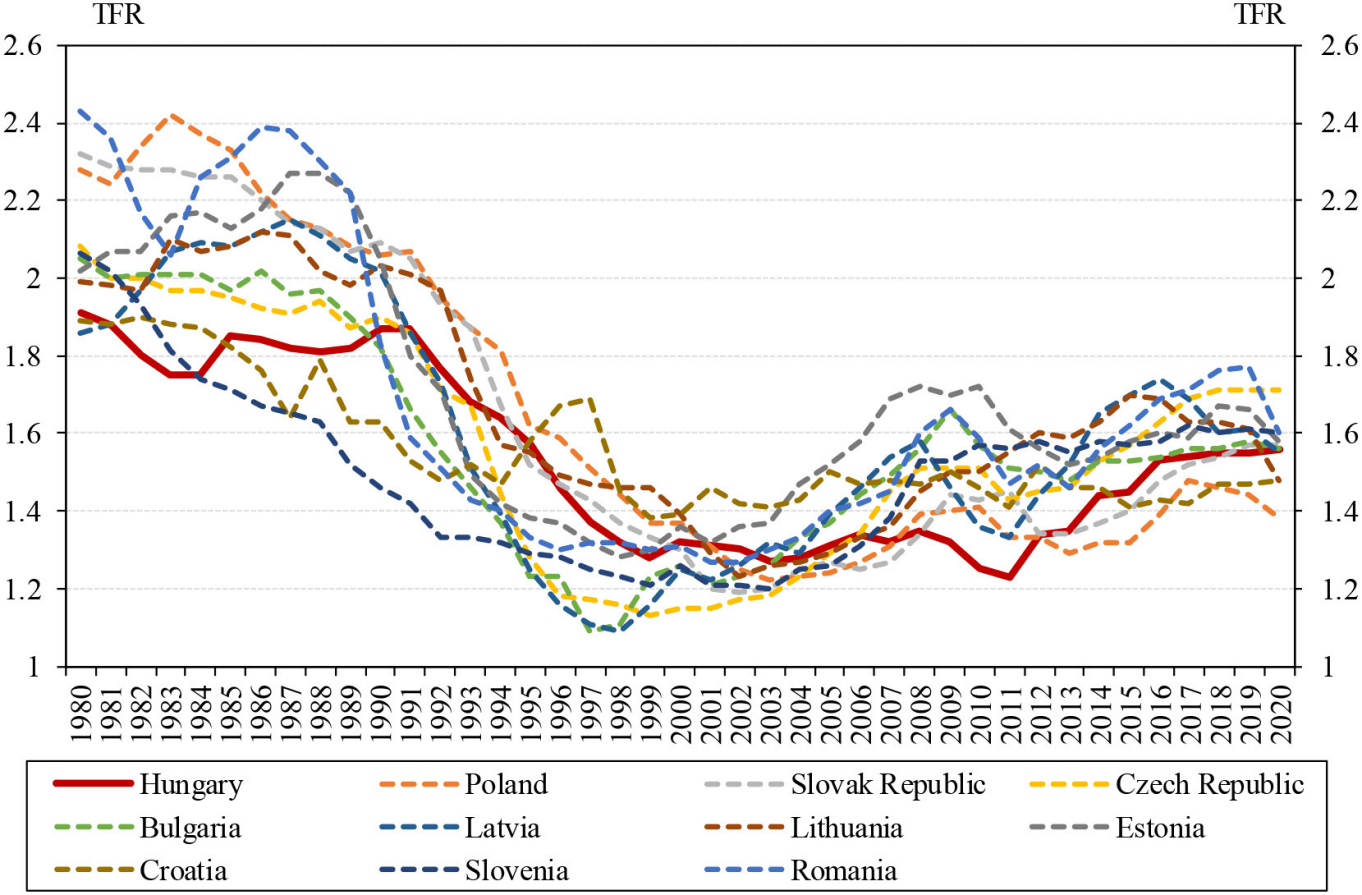
Trends in the total fertility rate of Central and Eastern European countries admitted to the European Union, 1980–2020. Source: World Bank (2022). Some countries in this figure are constituent republics of the former Yugoslavia. These countries may also have experienced a greater degree of political transition than other regime-changing countries. However, the degree of fertility transition experienced in these countries is roughly the same as in other regime-changing countries. It is true that Croatia did not witness the slight increase in TFR in recent years that most of the other countries did, but this also happened in Bulgaria and Estonia.

Our third argument for not overestimating the impact of family policy changes is based on a comparison with the fertility rates of the Central and Eastern European countries that joined the European Union. Figure 2 shows that the fertility of these countries has undergone similar changes since 1980 to those of Hungary. The slow decline in the rate seen in the 1980s after the regime change turned into a rapid fall until the early 2000s, followed by a slow rise or stagnation from then on. In 2020, the Hungarian total fertility rate was exactly the median of the region, where the Czech Republic, Romania, Slovenia, and Estonia have higher rates, Latvia, Lithuania, Poland, and Croatia lower, and the Bulgarian rates are broadly similar (1.58) (World Bank, 2022).

The data suggest that the increase in fertility in Central and Eastern Europe over the past decade is probably due to birth postponement. This is supported by the increases in the TFR between 2009 and 2017 in all the CEE countries studied, except for in Bulgaria, Estonia, and Croatia (Figure 2), which in many cases did not appear to be linked to family support programmes. We could not find a single database for family support programmes that covered all countries, but we can illustrate the situation with examples. In Poland, for example, public spending on family benefits increased from 1.8 to 3% of GDP between 2009 and 2017, while in the Czech Republic it decreased from 3.1 to 2.9% over the same period, but fertility increased by 0.08 in Poland and 0.18 in the Czech Republic. Similarly, Estonia and Latvia can be cited as examples: between 2009 and 2017 Estonia's share of family aid as a proportion of GDP increased by 0.17 percentage points, while Latvia's decreased by 0.19 percentage points, despite a 0.11 decrease in TFR in Estonia and a 0.23 increase in Latvia. Over the same period, family benefits as a share of GDP changed by +0.05 and −0.21 percentage points in Slovakia and Slovenia, while fertility increased by almost the same amount in both countries: 0.08 and 0.09, respectively (Eurostat, 2022; OECD, 2022).

**Figure 3:**
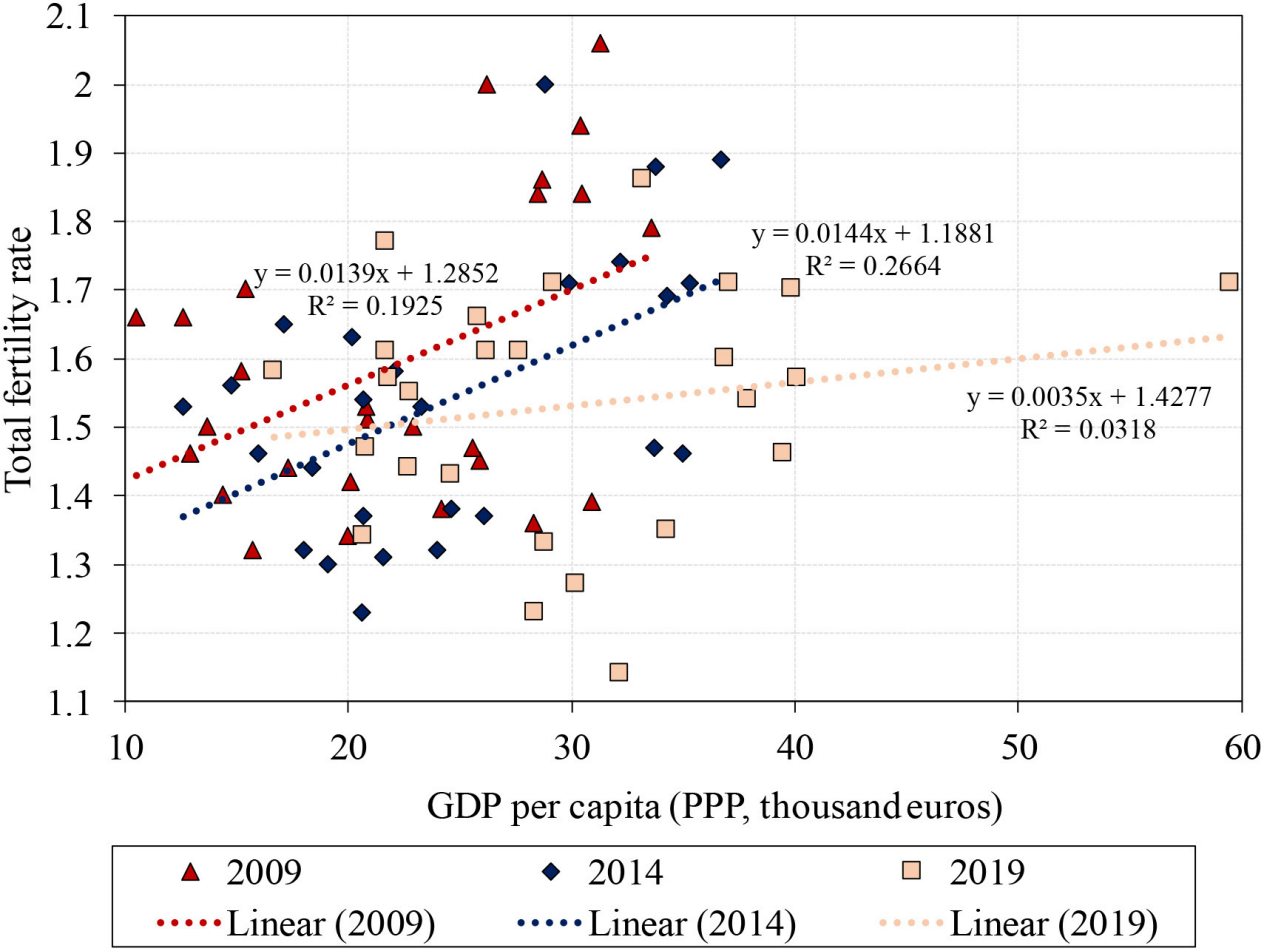
Relation between GDP per capita (purchasing power parity) and total fertility rate in the EU Member States. Source: Eurostat data downloaded on 5 June 2022 (Luxembourg was omitted as an outlier).

With regard to our fourth argument, on the effectiveness of Hungarian family policy support, we now refer to the TFR of older EU Member States. In the past, it was considered valid that after reaching a relatively high level of GDP, a further increase in GDP would help to increase the TFR (Myrskylä et al., 2009). This argument was supported by the fact that while in the 1990s the TFR in Southern Europe was very low, it showed an upward trend in Western and Northern Europe. However, later, Harttgen and Vollmer (2014), among others, pointed out that the perceived increase was not robust to later refined values of the Human Development Index, while Furuoka (2009) questioned in its entirety the existence of a positive correlation between development and fertility. In the light of more recent data, Gaddy (2021) has also reached a similar conclusion. The evolution of the correlation is illustrated in Figure 3.

Figure 3 shows the TFR values for GDP levels in the European Union countries at three points in time, 5 years apart. Luxembourg, with its high GDP and very low TFR, is classified as an outlier and is therefore excluded from the analysis. The linear trends, where GDP per capita is measured in thousands of euros, are fitted separately to the data 5 years apart and show an interesting correlation. The squares of the correlation coefficients (*R*^2^) of the linear trend lines are low at 0.1925, 0.2664, and 0.0318, in ascending order of years. Nevertheless, given the direction of the trend lines and the loose but nonetheless existing relationship, we can assume the following: Compared to 2009, in 2014 the trend line indicates lower TFR values with the same GDP levels and the slope of the trend line was also slightly less steep. In 2019, a linear line was obtained with a much smaller slope as compared to the 2014 trend, and the *R*^2^ value is very low. This indicates that two phenomena occurred over time. Firstly, higher GDP values were associated with lower fertility rates, and secondly, after a passage of time, it was less and less common for high GDP to be associated with a significantly higher TFR. The extremely low *R*^2^ in 2019 actually indicates that there was no longer a linear relationship between the GDP and the TFR. In other words, between 2009 and 2019 it was less and less true that richer countries in the European Union had higher fertility rates.

The underlying reason, in the view of the previously mentioned authors, is that while the less developed countries were still in the phase of childbirth postponement in the early 2000s, more developed countries were already catching up – recuperating – but with an older maternal age. This resulted in a higher per capita GDP and a higher TFR. However, by the end of the 2000s this catch-up period had ended, also sharply reducing the TFR in more developed countries. The lesson for us is the following: regardless of whether families become richer as a consequence of economic development or as a result of targeted family benefits, they are not inclined to have more children.

## Explaining the even greater decline in fertility rates than before – in the spirit of Becker

As described earlier, since Becker (1960), the decrease in TFR has been explained in the literature mainly as a trade-off between the quantity and quality of children. However, we believe that Becker's model in its basic form is insufficient to explain the drastic decline in TFR that we are seeing in developed countries today. Therefore, in this section, we reshape the initial assumptions of the Becker model somewhat so that the modified model can explain even very low TFR values.

Our analysis is theoretical in nature, providing a microeconomic model context to explain the facts presented in the previous sections. The central element of our model is the utility function for individuals. The utility function is a theoretical construct, explained in practice by the behavior of agents. In our case, this is precisely the reduction in the mother's propensity to give birth^3^.

The mathematical formulation of the exchange between quantity and quality of children has been expressed slightly differently by various authors. In our description, we rely mainly on Becker (1992), although we also modify the notation he uses to some extent. A similar line of thought can be found in the first paper of this kind by Becker (1960), and also in Becker and Lewis (1973) and Willis (1973), among others.

Hereafter, the term “family” is also used to refer to couples with children. It is assumed that contraceptive methods are available so that the birth of a child is intended. The decision is then made on the basis of the family's utility function – Equation (1) – and the family's budget constraints – Equation (2).

The utility function^4^ of the family:


(1) $$\begin{array}{l} U=v\left(x\right)+b\left(n,q\right)=U\left(x,n,q\right) \end{array}$$

Where x is the quantity of other goods consumed by the family; n is the number of children; and the quality of each child in the family is q. We assume that *b* is concave in *q* (although Becker, 1992 assumed only quasi-concavity).

The budget-related equation of the family:


(2) $$\begin{array}{l} p_{x}x+\left(p_{n}+p_{q}q\right)n=p_{x}x+\Pi_{c}n=I \end{array}$$

Where *p*_*x*_ is the fixed price of other goods; *p*_*n*_ refers to the cost of child-rearing not depending on quality; *p*_*q*_ refers to variable costs which depend on quality, like education, health, and other similar factors; and *Π*_*c*_ is the total cost of raising a child: i.e., *Π*_*c*_ = *p_n_* + *p*_*q*_. Finally, *n* is the number of children.

Becker and his followers considered the price associated with quality, *p*_*q*_, to be fixed within a family, similarly to the cost of raising children independent of quality, denoted as *p*_*n*_. So the price of a child, *Π*_*n*_, is also fixed. In reality, however, the price of children with higher parity (higher birth order) may decrease, be fixed, or increase. Examples of each of these situations are found in the literature. The intriguing question is how it can be that many families in more developed countries “buy” very few (e.g., one) children, or do not even produce a first child. This can be explained in the spirit of Becker (1992) in the following way:

If, in the case of given *q* child quality


(3) $$\begin{array}{l} U\left(x-\Pi_{c},1,q\right)<U\left(x,0,q\right), \end{array}$$

then no child is “bought”. Otherwise, a first child is “bought”. If


(4) $$\begin{array}{l} U\left(x-2\Pi_{c},2,q\right)<U\left(x-\Pi_{c},1,q\right), \end{array}$$

no second child is “bought”. Otherwise, one is. And so on.

If the price *Π*_*c*_ of a child depends on the number of children, then (3) and (4) may be modified as in equations (3′) and (4′). In the following, the first subscript index *c* of *Π*_*c,j*_ irefers to the total cost of child-rearing, and the second index denotes the birth order of the respective child:


(3′) $$\begin{array}{l} U\left(x-\Pi_{c,1},1,q\right)<U\left(x,0,q\right) \end{array}$$

(4′) $$\begin{array}{l} U\left(x-\Pi_{c,1}-\Pi_{c,2},2,q\right)<U\left(x-\Pi_{c,1},1,q\right) \end{array}$$

And so on.

Whether the ith child is “bought” depends on the price of the ith child and the shape of the utility function. If the utility function U(x, n, q) is not only weakly but strongly concave in its variables, as we now assume, then even with a decrease in “price”, families will still have a small number of children. The focus is on the existence of inequalities (3′) and (4′), and other similar inequalities. In such cases, even reducing the cost of having children through economic policy interventions has little effect on strengthening couples' intention to have children. Table 1 below summarizes how different conceptual approaches can be used to explain the declining propensity to have children.

**Table 1:** Conditions for a decrease in the propensity to have children according to different model concepts.

**Developers of the model (concept)**	**Cost of having children (*Π*_c_)**	**Marginal utility of a child**
E.g., Becker (1960), Becker and Lewis (1973), Willis (1973), Becker (1992), Mihályi (2019a) etc.	Same for all children within the family, but by adapting to external circumstances the family chooses the costs.	Decreasing.
E.g., Mihályi (2019b), Abendroth et al. (2014), Tan et al. (2016)	Cost of higher parity child (sibling born later) is higher.	Declining, but the decrease in childbearing intention can be explained even with a slight increase in marginal benefit.
E.g., Hirsch et al. (2012), Schnaiberg (1973), Holmes and Tiefenthaler (1997).	Cost of having children per family decreases per parity.	Very strongly decreasing.

Studies that suggest increases or decreases in child-rearing-related marginal costs do not necessarily assume increasing or decreasing marginal costs over the entire parity range, but they do make this assumption for some parties. All of the studies incorporate indirect child-rearing costs (in Becker's terminology, this means the costs in terms of quality); some focus on them directly, but the methods of calculation are not uniform.

In Table 1, the last row is particularly noteworthy from an economic policy perspective. Various state benefits in cash and in kind can be used to reduce the cost of first and higher-parity children. However, if the marginal utility of families declines sharply with the number of children, economic policy measures will not achieve their goal. In our view, this is exactly what is happening in most European countries today.

## Conclusion

We have shown, using the example of Hungary and to some extent those of the countries of the European Union, that the TFR has almost never reached the replacement rate in recent decades. This will inevitably cause a population decline, and in some countries such as Hungary the population is already decreasing. Such a decrease can only be prevented by welcoming large numbers of immigrants, which may cause other kinds of problems.

If the fertility rate are to rise permanently above 2.1, and emigration were not to reduce a country's population, population decline might be halted again many years in the future. This is why Hungary and many other European countries have introduced various family-support measures. However, these measures have produced only modest results to date. In order for such measures to be effective, the preferences of families (couples) will need to change. In addition to the options offered by contraception, it is useless to reduce the cost of child-rearing if the next child in parity order does not sufficiently increase the utility of the family. This idea is clearly explained by the Beckerian trade-off between quantity and quality. However, with a drastic reduction in the TFR, the choices of families cannot always be traced back to the original Becker model. Thus, a further tightening of the model is required. Assuming that the utility function for child-bearing is concave instead of the quasi-concavity assumed by Becker provides an acceptable explanation of why families do not want children.

Since there is no indication of forthcoming modifications in the preferences of families, society will be required to adapt to the situation. It is unnecessary to increase the already high level of family-support expenditure, a significant part of which will be absorbed through other channels in any case. We must recognize the aging of the population and concentrate available resources on ensuring that, despite this aging process, all citizens enjoy a decent quality of life. This requires the organization of age-friendly jobs, the provision of appropriate medical care, and ensuring the financial security of very old people. There is also room to improve the education system for young people and provide them with more human capital. This investment in human capital will help them to cope with the aging phase of their lives better as society really does become older. However, these issues are subjects for another article.

## Data availability statement

The original contributions presented in the study are included in the article, further inquiries can be directed to the corresponding author.

## Author contributions

ÉB and ÁD contributed equally to the article. ÉB has largely contributed the chapter on the further development of the Becker model, while ÁD has largely provided the framework for the chapter. Some examples of measures taken to increase fertility rates. All authors contributed to the article and approved the submitted version.

## Funding

This work was supported by the Cooperative Doctoral Program (Kooperatív Doktori Program) of the Ministry for Innovation and Technology and National Research, Development, and Innovation Office.

## Conflict of interest

The authors declare that the research was conducted in the absence of any commercial or financial relationships that could be construed as a potential conflict of interest. The reviewer FB declared a past co-authorship with the author ÉB to the handling editor.

## Publisher's note

All claims expressed in this article are solely those of the authors and do not necessarily represent those of their affiliated organizations, or those of the publisher, the editors and the reviewers. Any product that may be evaluated in this article, or claim that may be made by its manufacturer, is not guaranteed or endorsed by the publisher.

## Footnotes

^1^In developed countries, the fertility rate associated with the reproductive level is around 2.1, but in less developed countries it is higher, in some cases exceeding 2.5 due to higher mortality rates (Gietel-Basten and Scherbov, 2020).

^2^See https://miniszterelnok.hu/orban-viktor-beszede-a-magyar-diaszpora-tanacs-viii-ulesen/ (downloaded 15 Jun 2022).

^3^In our modeling, we use the following approach: “All models are wrong, but some are useful” (Box, 1979), and try to explain the observed events in theoretical contexts. Our model helps us to identify the causes of declining TFR and points out that we can only expect a strengthening of the intention to have children if preferences change.

^4^The utility function was used in accordance with the conceptual framework of neoclassical microeconomics, in the same way that Becker (1960) used it, to quantify the value judgements of families.

## References

[B1] Aarts O. Need A. Grotenhuis M. Graaf N. D. (2008). Does belonging accompany believing? Correlations and trends in Western Europe and North America between 1981 and 2000. Rev. Relig. Res. 50, 16–34.

[B2] Abendroth A. K. Huffman M. L. Treas J. (2014). The parity penalty in life course perspective: motherhood and occupational status in 13 European countries. Am. Sociol. Rev. 79, 993–1014. 10.1177/0003122414545986

[B3] Angrist J. Lavy V. Schlosser A. (2010). Multiple experiments for the causal link between the quantity and quality of children. J. Lab. Econ. 28, 773–824. 10.1086/653830

[B4] Atoyan R. Christiansen L. Dizioli A. Ebeke C. Ilahi N. Ilyina A.. (2016). Emigration and its economic impact on eastern Europe. IMF Staff Discussion Note. Available online at: https://www.imf.org/external/pubs/ft/sdn/2016/sdn1607.pdf (accessed May 15, 2022).

[B5] Becker G. S. (1960). An Economic Analysis of Fertility. Demographic and Economic Change in Developed Countries. New York, NY: Columbia University Press, 209–240.

[B6] Becker G. S. (1986). An Economic Analysis of the Family. Dublin: Economic and Social Research Institute.

[B7] Becker G. S. (1992). Fertility and the economy. J. Pop. Econ. 5, 185–201. 10.1007/BF00172092 12285413

[B8] Becker G. S. Lewis H. G. (1973). On the interaction between the quantity and quality of children. J. Polit. Econ. 81, S279–S288. 10.1086/26016612178275

[B9] Becker G. S. Lewis H. G. (1974). Interaction between quantity and quality of children, in Economics of the Family: Marriage, Children, and Human Capital, ed Schultz T. W. (Chicago, IL: University of Chicago Press), 81–90.

[B10] Ben-Porath Y. (1973). Economic analysis of fertility in Israel: point and counterpoint. J. Polit. Econ. 81, S202–S233. 10.1086/260162

[B11] Blake J. (1981). Family size and the quality of children. Demography 18, 421–442. 10.2307/2060941 7308532

[B12] Bloom D. Canning D. Fink G. (2010). Implications of population ageing for economic growth. Oxford Rev. Econ. Policy 26, 583–612. 10.1093/oxrep/grq038

[B13] Bloom D. Canning D. Sevilla J. (2003). The Demographic Dividend: A New Perspective on the Economic Consequences of Population Change. Arlington, VA: Rand Corporation.

[B14] Bongaarts J. (1982). The fertility-inhibiting effects of the intermediate fertility variables. Stud. Family Plann. 179–189. 10.2307/1965445 7112629

[B15] Bongaarts J. Feeney G. (1998). On the quantum and tempo of fertility. Pop. Dev. Rev. 24, 271–291. 10.2307/2807974

[B16] Box G. E. (1979). All models are wrong, but some are useful. Robustness Stat. 202, 1549.

[B17] Caldwell J. C. (1978). A theory of fertility: from high plateau to destabilization. Pop. Dev. Rev. 4, 553–577. 10.2307/1971727

[B18] Clark T. C. (1969). Religion, morality, and abortion: a constitutional appraisal. Loy. ULAL Rev. 2, 1.

[B19] Davie G. (1994). Religion in Britain Since 1945: Believing Without Belonging. Oxford: Blackwell.

[B20] Doepke M. (2005). Child mortality and fertility decline: does the Barro-Becker model fit the facts? J. Pop. Econ. 18, 337–366. 10.1007/s00148-004-0208-z

[B21] Ehrlich P. R. (1968). The population bomb. Think. About Environ. 24, 72–80.

[B22] Eurostat (2022). [database]. Available online at: https://ec.europa.eu/eurostat/data/database (accessed April 15, 2022).

[B23] Furuoka F. (2009). Looking for a J-shaped development-fertility relationship: do advances in development really reverse fertility declines. Econ. Bullet. 29, 3067–3074.

[B24] Gaddy H. G. (2021). A decade of TFR declines suggests no relationship between development and sub-replacement fertility rebounds. Demograph. Res. 44, 125–142. 10.4054/DemRes.2021.44.5

[B25] Gietel-Basten S. Scherbov S. (2020). Exploring the ‘true value’of replacement rate fertility. Pop. Res. Policy Rev. 39, 763–772. 10.1007/s11113-019-09561-y

[B26] Goldstein J. R. Cassidy T. (2021). The formal demography of peak population. Ext. Abstract PAA. Available online at: https://www.oeaw.ac.at/fileadmin/subsites/Institute/VID/PDF/Conferences/2021/Slides/Keynote_Goldstein_extended_abstract.pdf (accessed May 5, 2022).

[B27] Harttgen K. Vollmer S. (2014). A reversal in the relationship of human development with fertility? Demography 51, 173–184. 10.1007/s13524-013-0252-y 24197749

[B28] Häyry M. (2004). If you must make babies, then at least make the best babies you can? Hum. Fertility 7, 105–112. 10.1080/14647270410001699063 15223759

[B29] HFD (2022). Human Fertility Database. Max Planck Institute for Demographic Research (Germany) and Vienna Institute of Demography (Austria). Available online at www.humanfertility.org (accessed May 15, 2022).

[B30] Hirsch D. Sutton L. Beckhelling J. (2012). The Cost of a Child in the Twenty-First Century. London: Child Poverty Action Group London.

[B31] Holmes J. Tiefenthaler J. (1997). Cheaper by the dozen? The marginal time costs of children in the Philippines. Pop. Res. Policy Rev. 16, 561–578. 10.1023/A:1005756622764

[B32] Kamarás F. (2010). Népesedési Helyzet. KSH Társadalmi Helyzetkép. Nové Zámky: Magyar Központi Statisztikai Hivatal.

[B33] Lutz W. (2005). Policies addressing the tempo effect in low-fertility countries. Pop. Dev. Rev. 31, 699–720. 10.1111/j.1728-4457.2005.00094.x

[B34] Lutz W. (2007). The future of human reproduction: will birth rates recover or continue to fall? Ageing Horizons 7, 15–21.

[B35] Magyar Nemzeti Bank (2022). Housing Market Report. Available Online at: https://www.mnb.hu/letoltes/laka-spiaci-jelente-s-2022-ma-jus-eng.pdf (accessed May 15, 2022).

[B36] Malmberg B. Tamas K. Bloom D. Munz R. Canning D. (2006). Global Population Ageing, Migration and European External Policies. Stockholm: Institute for Future Studies.

[B37] Malthus T. (1798). An Essay on the Principle of Population: An Essay on the Principle of Population as it Affects the Future Improvment of Society with Remarks on the Speculations of Mr. Godwin, M. Condorcet, and Other Writers. St. Paul's Church-Yard: Springer.

[B38] McDonald P. (2006). Low fertility and the state: the efficacy of policy. Pop. Dev. Rev. 32, 485–510. 10.1111/j.1728-4457.2006.00134.x

[B39] Mihályi P. (2019a). Marginal utilities and marginal costs of having children. Pub. Financ. Q. 64, 526–541. 10.35551/PFQ_2019_4_5

[B40] Mihályi P. (2019b). A gyermekvállalás határhasznai és határköltségei mikro-, mezo- és makroszinten. Demográfia 62, 311–345. 10.21543/Dem.62.4.1

[B41] Molteni F. Biolcati F. (2018). Shifts in religiosity across cohorts in Europe: a multilevel and multidimensional analysis based on the european values study. Soc. Compass 65, 413–432. 10.1177/0037768618772969

[B42] Myrskylä M. Kohler H. P. Billari F. C. (2009). Advances in development reverse fertility declines. Nature 460, 741–743. 10.1038/nature08230 19661915

[B43] Notestein F. W. (1953). Economic Problems of Population Change. London: Oxford University Press.

[B44] OECD (2022). OECD Family Database—OECD. Available online at: https://www.oecd.org/els/family/database.htm(accessed May 15, 2022).

[B45] Oláh L. S. (2003). Gendering fertility: second births in Sweden and Hungary. Pop. Res. Policy Rev. 22, 171–200. 10.1023/A:1025089031871

[B46] Parr N. (2021). Which of Europe's migration-receiving countries face long run population decline?, Paper Presented to the Wittgenstein Centre Conference on Causes and Consequences of Depopulation (Vienna).

[B47] Pollack D. (2008). Religious change in Europe: Theoretical considerations and empirical findings. Soc. Compass 55, 168–186. 10.1177/0037768607089737

[B48] Rosenzweig M. R. Evenson R. (1977). Fertility, schooling, and the economic contribution of children of rural India: an econometric analysis. Econ. J. Econ. Soc. 1:1065–1079. 10.2307/1914059 12278790

[B49] Schnaiberg A. (1973). The concept and measurement of child dependency: an approach to family formation analysis. Pop. Stu. 27, 69–84. 10.1080/00324728.1973.10410314 22074198

[B50] Schultz T. W. (1973). The value of children: an economic perspective. J. Polit. Econ. 81, S2–S13. 10.1086/260151

[B51] Sobotka T. (2004). Is lowest-low fertility in Europe explained by the postponement of childbearing? Pop. Dev. Rev. 30, 195–220. 10.1111/j.1728-4457.2004.010_1.x

[B52] Spéder Z. (2021). Termékenységi mintaváltás–a családalapítás átalakulásának demográfiai nyomvonalai magyarországon fertility pattern change–demographic traces of the transformation of family formation in Hungary. Szocioló. Szemle 31, 4–29. 10.51624/SzocSzemle.2021.2.1

[B53] Spéder Z. Kamarás F. (2008). Secular fertility decline with distinct period fluctuations. Demo. Res. 19, 599–664. 10.4054/DemRes.2008.19.18

[B54] Spéder Z. Kapitány B. (2014). Failure to realize fertility intentions: a key aspect of the post-communist fertility transition. Pop. Res. Policy Rev. 33, 393–418. 10.1007/s11113-013-9313-6

[B55] Szikra D. Szelewa D. (2010). Do central and eastern European countries fit the “Western” picture? The example of family policies in Hungary and Poland, in Welfare States and Gender Inequality in Central and Eastern Europe, eds Klenner C. Leiber S. (Brussels: European Trade Union Institute), 81–114.

[B56] Tan P. L. Morgan S. P. Zagheni E. (2016). A case for “reverse one-child” policies in Japan and South Korea? Examining the link between education costs and lowest-low fertility. Pop. Res. Policy Rev. 35, 327–350. 10.1007/s11113-016-9390-4 29593367 PMC5869025

[B57] United Nations (2022). World Population Prospects 2022. Available Online at: https://population.un.org/wpp/ (accessed May 15, 2022).

[B58] Vollset S. E. Goren E. Yuan C. W Cao J. Smith A. E.. (2020). Fertility, mortality, migration, and population scenarios for 195 countries and territories from 2017 to 2100: a forecasting analysis for the global burden of disease study. Lancet 396, 1285–1306. 10.1016/S0140-6736(20)30677-2 32679112 PMC7561721

[B59] Willis R. J. (1973). A new approach to the economic theory of fertility behavior. J. Polit. Econ. 81, S14–S64. 10.1086/260152

[B60] World Bank (2022). Fertility rate, total (births per woman) Data. Available online at: https://data.worldbank.org/indicator/SP.DYN.TFRT.IN (accessed May 15, 2022).

[B61] Zhang L. (2008). Religious affiliation, religiosity, and male and female fertility. Demographic 18, 233–262. 10.4054/DemRes.2008.18.8

